# Hormonal and metabolic indicators before and after farrowing in sows affected with postpartum dysgalactia syndrome

**DOI:** 10.1186/s12917-018-1649-z

**Published:** 2018-11-07

**Authors:** Marianne Kaiser, Stine Jacobsen, Pia Haubro Andersen, Poul Bækbo, José Joaquin Cerón, Jan Dahl, Damián Escribano, Peter Kappel Theil, Magdalena Jacobson

**Affiliations:** 1 Department of Veterinary Clinical Sciences, Faculty of Health and Medical Sciences, University of Copenhagen, Agrovej 8, 2630 Taastrup, Denmark; 2 Faculty of Veterinary Medicine and Animal Science, Department of Clinical Sciences, Swedish University of Agricultural Sciences, P.O. Box 7054, SE-750 07 Uppsala, Sweden; 3 SEGES, Danish Pig Research Centre, Agro Food Park 15, 8200 Aarhus N, Denmark; 4 Department of Animal Medicine and Surgery, Regional “Campus of Excellence Mare Nostrum”, University of Murcia, Espinardo, 30100 Murcia, Spain; 5 Danish Agriculture and Food Council, Axelborg, Axeltorv 3, 1709 Copenhagen V, Denmark; 6 Department of Animal Science - Molecular nutrition and reproduction, Aarhus University, Blichers Allé 20, 8830 Tjele, Denmark

**Keywords:** PPDS, Chromogranin A, Prostaglandin F2α, Glucose metabolism, Cortisol

## Abstract

**Background:**

Postpartum dysgalactia syndrome (PDS) in sows is difficult to diagnose and the pathogenesis is obscure. Hormonal changes related to the disease are often difficult to distinguish from those found in the normal transition period from gestation to lactation. The study aimed to investigate metabolic and hormonal changes related to PDS with the goal of identifying potential biomarkers in sows suffering from PDS (PDS+). Selected biomarkers were examined by comparing 38 PDS+ sows with 38 PDS negative (PDS-) sows. The sows were sampled every 24 h from 60 h *ante partum* (*a.p.*) to 36 h post partum *(p.p.)*.

**Results:**

Compared to the baseline (60 to 36 h *a.p.*), cortisol in serum and saliva and fasting blood glucose concentrations increased in PDS+ as well as PDS- sows. C-peptide decreased relative to the baseline in PDS+ sows, and prolactin and 8-epi prostaglandin F2 alpha (8-epi-PGF2α) decreased in PDS- sows. Concentrations of cortisol in serum and saliva, salivary chromogranin A (CgA), fasting blood glucose, C-peptide, and 8-epi-PGF2α differed significantly between PDS+ and PDS- sows, with levels of cortisol in serum and saliva, salivary CgA, and 8-epi-PGF2α in serum being different in the two groups already before parturition. Concentrations of salivary CgA were significantly lower in PDS- sows than in PDS+ sows during the entire study period.

**Conclusions:**

The results suggest that salivary CgA, cortisol and serum 8-epi-PGF2α may potentially serve as early diagnostic indicators for PDS. The consistently higher salivary CgA concentration in PDS+ sows compared to PDS- sows may indicate that homeostatic disturbances are present between 36 to 60 h before parturition in sows developing PDS. The higher serum and saliva cortisol concentration in PDS+ sows compared to PDS- sows could reflect an early sign of inflammation or stress. The significantly lower C-peptide in PDS+ sows compared to PDS- sows may reflect a lower food intake. Our results contribute to the understanding of the pathogenesis of PDS, and the homeostatic disturbances detected before parturition warrants further investigation. The diagnostic potential of the markers identified in this study should be investigated further in a larger population of sows.

**Electronic supplementary material:**

The online version of this article (10.1186/s12917-018-1649-z) contains supplementary material, which is available to authorized users.

## Background

The pathogenesis of postpartum dysgalactia syndrome (PDS) in sows is not fully understood, and the clinical diagnosis is often difficult. Commonly used indicators for PDS include fever, reduced appetite, mastitis, and signs of piglet starvation. These signs, however, may vary considerably [1–4]. Improved knowledge about the pathogenesis of PDS, including disturbances in hormonal and metabolic processes at parturition, may thus be relevant for future diagnosis, treatment and prophylaxis.

In the transition from gestation to lactation, mammals undergo extensive hormonal changes [5, 6], including a rapid shift from an anabolic to a catabolic state [6]. Serum concentrations of progesterone and estradiol decrease, and cortisol concentrations increase temporarily [7]. Prolactin concentrations starts to increase close to parturition and increase further at the onset of lactation [8, 9]. Glucose is the most important nutrient for milk production [10, 11], as it is a precursor for lactose [12]. Cortisol serves to mobilize glucose from glycogen stores [13, 14], and the peripartal cortisol release [15, 16] thus leads to increased blood glucose levels [17] in healthy sows. A number of other biological events are also associated with increased levels of cortisol, for example stress [18] and inflammation [19, 20].

Factors like inadequate body condition of the sow [21, 22], improper feeding strategy in late gestation [23], or improper feed composition [22, 24] may negatively affect the production of colostrum and milk, thus limiting piglet growth and survival [25]. Reduced appetite and feed intake are normal peri-parturient features in healthy sows [26], but excessive anorexia may be a sign of illness. Decreased feed intake decreases glucose uptake from the gastrointestinal tract, but under normal circumstances adequate blood glucose levels are also maintained in sows ingesting less feed than intended [27]. C-peptide concentrations reflect the insulin response to glucose [28], and the excretion is correlated to insulin [29]. Measuring C-peptide concentrations instead of insulin is advantageous, since C-peptide is more stable [30, 31]. C-peptide has not been investigated in sows before.

Cromogranin A (CgA) reflects activation of the sympatho-adrenal medullary system (SAM) [32, 33] and is secreted into saliva. It is necessary for the regulation of vascular homeostasis in humans [34] and concentrations are increased in individuals with neuroendocrine tumors [35] and endocrine cells of the human gastrointestinal tract [36, 37]. In humans, CgA levels can be elevated by hypertension, inflammatory bowel disease, sepsis, and other inflammatory diseases [34]. It has been linked to oxidative stress in humans [38] and shown to be a trigger of free radical production in rats [39]. CgA has only been studied to a limited extent in pigs, but it was recently shown by Escribano and coworkers [40] to be a marker of stress in a pig model.

Eight-epi prostaglandin F2 alpha (8-epi-PGF2α) is considered to be a biomarker of oxidative stress in humans [41], and it is released when free oxygen radicals are produced in excess or when antioxidants are lacking [42]. Vitamin E and selenium deficiencies have been shown to inhibit the immune function in sows [43], and antioxidants seem to reduce the risk of sow agalactia [44]. To our knowledge, 8-epi-PGF2α has not been studied in sows before.

The aims of this study were to describe the changes of cortisol, CgA, glucose, C-peptide, prolactin, 8-epi-PGF2α, sodium, and potassium levels in healthy sows (PDS-) and sows suffering from PDS (PDS+) during the periparturient period. Furthermore, we aimed to evaluate the potential of these biomarkers to identify affected animals in the early stage of disease.

## Results

### Body condition score

A body condition score of 2 was noted in 12 PDS+ sows and 17 PDS- sows, and score 3 was assigned to 26 PDS+ sows and 21 PDS- sows (*p* = 0.09).

### Obstetric aid, parturition duration and feeding time

Obstetric aid was provided more often in PDS+ sows (18 PDS+ sows and 11 PDS- sows; *p* < 0.05) and parturition lasted longer for PDS+ sows (mean length 654.2 min; SD 444.0) compared to PDS- sows (mean length 432.3 min; SD 267.5) (*p* < 0.01). Feeding time relative to parturition (less than or more than 4 h before parturition) had no influence on whether sows were categorized as PDS+ sows or PDS- sows (*p* = 0.16).

### Piglet weight gain

**Fig. 1:**
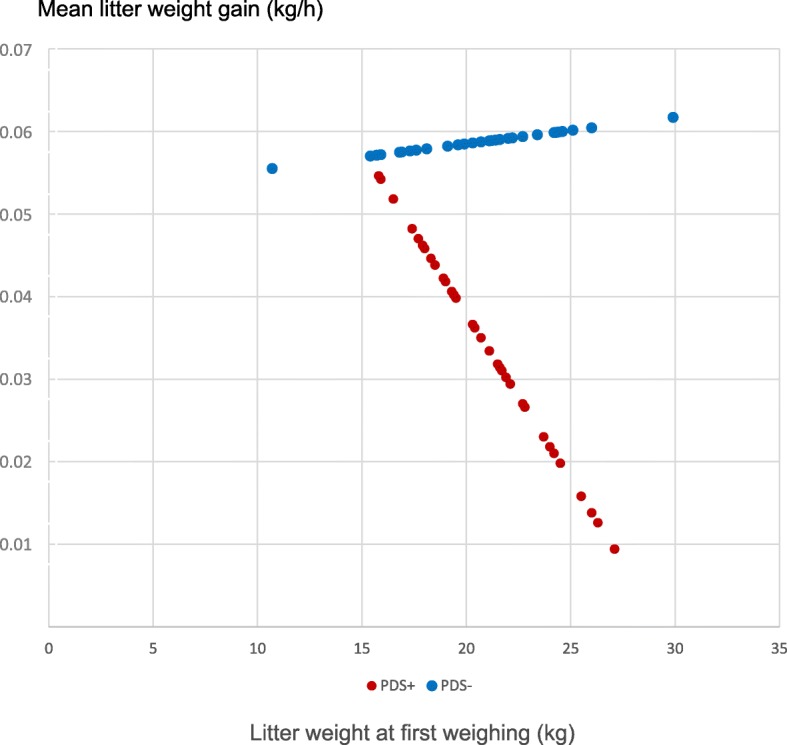
Mean weight gain (kg/h) in litters from 38 PDS+ sows and litters from 38 PDS- sows. In PDS+ sows, the litter weight gain depended on the litter weight (kg) at first weighing, where litters with the highest weight had the smallest weight gain. This interdependency was not apparent in litters born to PDS- sows

The effect of PDS status on litter weight gain was dependent on litter weight at first weighing (a significant interaction was noted between PDS status and weight at first weighing; *p* < 0.01). For the PDS+ sows, the litter weight gain decreased with increasing litter weight at first weighing, but for the PDS- sows, the litter weight gain was not dependent on weight at first weighing (Fig. 1).

### Changes in hormonal and metabolic indicators in relation to parturition

**Fig. 2:**
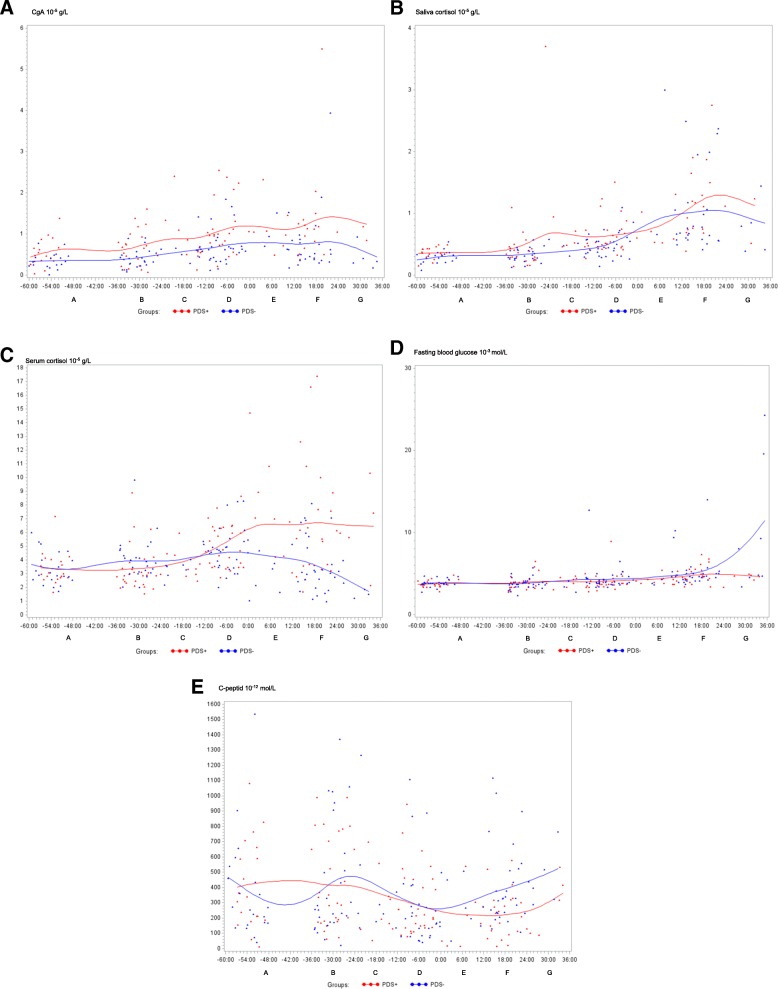
Raw data for A. chromogranin A (CgA; 10^− 5^ g/L), B. saliva cortisol (10^− 5^ g/L), C. serum cortisol (10^− 5^ g/L), D. fasting blood glucose (10^− 3^ mol/L), E. C-peptide (10^− 12^ mol/L) and F. 8-epi prostaglandin F2 alpha (8-epi-PGF2α; 10^− 9^ g/L) assessed from 60 h *ante partum* (time interval A) until 36 h post partum (time interval G) in sows suffering from postpartum dysgalactia syndrome (PDS+, red) and healthy sows (PDS-, blue). Each point represents the precise sample time of each observation relative to parturition of piglet number one (0 h). The line represents the mean value at each sampling time point

Changes over time for PDS+ and PDS- sows are illustrated by raw data in Fig. 2 for CgA (Fig. 2A), saliva cortisol (Fig. 2B), serum cortisol (Fig. 2C), fasting blood glucose (Fig. 2D), C-peptide (Fig. 2E) and 8-epi-PGF2α (Fig. 2F).

When compared to baseline A (60 to 36 h *a.p.*), hormonal and metabolic changes occurred over time in both PDS+ and PDS- sows. Differences between time interval A and intervals B to G are reported with lowercase letters in Table 1; In PDS+ sows, cortisol (serum and saliva) and fasting blood glucose increased significantly over time (B to G) relative to baseline A, while C-peptide decreased over time. In PDS- sows, increases over time in cortisol (serum and saliva) and fasting blood glucose concentrations were also noted relative to the  baseline. In contrast to PDS+ sows, serum cortisol levels in PDS- sows were only significantly increased relative to the baseline at − 12 to 0 h *p.p*, and significantly decreased 24 to 36 h *p.p.* Compared to the baseline, no significant changes were noted for C-peptide in PDS- sows. Concentrations of prolactin (Additional file 1) and 8-epi-PGF2α decreased significantly after parturition in PDS- sows but remained unaltered in PDS+ sows (Table 1). Concentrations of Na and K did not change over time in any of the groups (Additional files 2 and 3).

**Table 1:** Changes in concentrations of hormones and metabolic biomarkers in 38 sows suffering from postpartum dysgalactia syndrome (PDS+) and 38 healthy (PDS-) sows

Parameter	Group	*n*	A. (−60 to − 36 h)		B.(− 36 to − 24 h)		C. (− 24 to − 12 h)		D. (− 12 to 0 h)		E. (0 to 12 h)		F. (12 to 24 h)		G. (24 to 36 h)	
			LSMEANS	±SD	LSMEANS	±SD	LSMEANS	±SD	LSMEANS	±SD	LSMEANS	±SD	LSMEANS	±SD	LSMEANS	±SD
Serum cortisol	PDS+	38	3.14	1.10	**3.07** ^*^	**1.08**	3.34	1.15	4.84 ^**b**^	1.08	**6.36** ^***a^	**1.15**	**5.55** ^****a^	**1.09**	**5.43** ^***c^	**1.19**
	PDS-	38	3.18	1.10	**3.89** ^*^	**1.09**	3.63	1.15	4.58 ^**c**^	1.08	**2.79** ^***^	**1.17**	**3.14** ^****^	**1.09**	**2.12** ^***d^	**1.19**
Saliva cortisol	PDS+	38	0.35	1.12	0.37	1.10	0.50	1.16	**0.61** ^*b^	**1.10**	0.71 ^**c**^	1.21	1.09 ^**a**^	1.13	0.82 ^**d**^	1.39
	PDS-	38	0.28	1.11	0.32	1.10	0.44 ^**d**^	1.16	**0.44** ^*c^	**1.10**	0.84 ^**a**^	1.19	0.95 ^**a**^	1.11	0.66 ^**b**^	1.23
^**†**^ **Chromogranin A** ^***^	**PDS+**	**38**	**0.39**	**1.19**	**0.46**	**1.16**	**0.68**	**1.25**	**0.98**	**1.16**	**0.97**	**1.31**	**1.07**	**1.19**	**1.18**	**1.62**
	**PDS-**	**38**	**0.26**	**1.18**	**0.35**	**1.16**	**0.58**	**1.25**	**0.56**	**1.15**	**0.70**	**1.28**	**0.61**	**1.17**	**0.34**	**1.37**
Fasting blood glucose	PDS+	38	3.76	0.37	3.91	0.36	4.07	0.50	4.10	0.36	4.53	0.49	4.89 ^**d**^	0.40	**4.64** ^****^	**0.69**
	PDS-	38	3.85	0.37	3.74	0.35	4.76	0.60	4.09	0.35	5.35 ^**d**^	0.57	4.98 ^**d**^	0.36	**9.55** ^****a^	**0.63**
C-peptid	PDS+	38	301.9	1.21	326.42	1.17	222.29	1.33	240.95	1.18	109.22 ^**c**^	1.35	**161.14** ^**d^	**1.20**	220.37	1.42
	PDS-	38	267.25	1.20	285.60	1.18	303.87	1.33	182.97	1.18	238.13	1.35	**349.32** ^**^	**1.18**	412.03	1.41
Prolactin	PDS+	38	11,553.00	1.13	12,358.51	1.11	12,891.20	1.18	12,356.04	1.11	9075.19	1.18	11,896.50	1.12	7873.04	1.22
	PDS-	38	12,610.69	1.12	13,355.72	1.11	12,187.91	1.18	12,410.52	1.11	11,292.57	1.18	11,443.76	1.12	8304.06 ^**d**^	1.21
8-epi prostaglandin F2 α	PDS+	38	41.71	4.91	40.78	4.08	44.02	7.18	**42.60** ^*^	**4.19**	31.87	7.15	38.41	4.62	25.37	8.75
	PDS-	38	44.39	4.70	38.10	4.12	44.07	7.06	**30.07** ^*d^	**4.09**	34.39	7.42	30.23 ^**d**^	4.28	29.36	8.53
Natrium	PDS+	38	148.58	1.02	148.68	1.01	150.63	1.02	147.00	1.01	149.64	1.02	153.62	1.02	152.23	1.03
	PDS-	38	147.41	1.02	151.64	1.01	144.68	1.03	151.40	1.01	148.96	1.03	151.28	1.01	155.18	1.03
Potassium	PDS+	38	4.47	1.03	4.55	1.03	4.35	1.05	4.34	1.03	4.69	1.05	4.50	1.03	4.27	1.06
	PDS-	38	4.37	1.03	4.53	1.03	4.24	1.04	4.45	1.03	4.47	1.05	4.44	1.03	4.89	1.05

Significant differences for each time interval are given as least-squares means (LSMEANS) and standard deviations (SD). Lowercase letters represent the level of significance between time interval A and the following time intervals (B to G, respectively): ^a^ *p* < 0.0001, ^b^ *p* < 0.001, ^c^ *p* < 0.01, ^d^ *p* < 0.05. Bold letters and asterisk symbols represent the level of significance between PDS+ and PDS- sows: ^****^ *p* < 0.0001, ^***^ *p* < 0.001, ^**^ *p* < 0.01, ^*^ *p* < 0.05

^†^No significant interaction between *time* and *case-control* was found for chromogranin A. Therefore, an all over significant difference for chromogranin A between PDS+ and PDS- sows (*p* < 0.001) was estimated using the statistic model B (OUTCOME PARAMETER_ij_ = μ + TIME_i_ + GROUP_j_ + ε)

### Differences between hormonal and metabolic parameters in PDS+ and PDS- sows

Concentrations of cortisol (serum and saliva), CgA, fasting blood glucose, C-peptide and 8-epi-PGF2α differed between PDS- and PDS+ sows (bold letters and asterisk symbols; Table 1). From − 36 to − 24 h, serum cortisol concentration was significantly lower in the PDS+ sows compared to the PDS- sows, while concentrations were significantly higher in the PDS+ sows compared to the PDS- sows 0–36 h *p.p*. Before parturition, significantly higher concentrations of salivary cortisol and 8-epi-PGF2α were demonstrated in the PDS+ sows (− 12 h to 0 h) compared to the PDS- sows. CgA concentration was significantly higher in the PDS+ sows compared to the PDS- sows throughout the whole study period. Fasting blood glucose was significantly lower at 24–36 h *p.p*. and C-peptide was significantly lower at 12–24 h *p.p*. in the PDS+ sows compared to the PDS- sows (Table 1).

## Discussion

To our knowledge, this is the first study that compares hormonal and metabolic alterations in sows suffering from PDS and healthy sows in the immediate periparturient period.

Previously established criteria were selected to identify sows suffering from PDS [4, 45–51], but as already pointed out by others [52–54], these vary greatly and are associated with uncertainty. However, the present study, and previous results showing significant differences between PDS+ and PDS- sows for several inflammatory parameters [55] support the usefulness of our PDS definition.

Lactogenesis is influenced by local mammary factors (e.g. hormonal receptors), by the removal of milk through suckling, and by circulating nutrients and hormones [56]. Weight loss was more pronounced in heavy litters, which are expected to suckle with most intensity, and the limiting factor for milk production in PDS+ sows was thus most likely related to insufficient nutrient supply or hormonal disturbances.

Obstetric aid was more frequently given in sows diagnosed with mastitis, metritis and agalactia [57], coliform mastitis [58] and post-farrowing discharge [59], but the significance of the parturition duration in PDS is obscure [4, 50, 57]. Whether extended parturition and increased obstetric aid cause PDS or vice versa remains to be elucidated.

### Chromogranin A and cortisol

The continuously increased CgA concentration among the PDS+ sows at all time intervals may indicate a disturbance in the homeostasis of these sows. Interestingly, this disturbance seemed to occur before the systemic inflammation that, based on clinical signs and blood biochemical changes, became evident from 12 to 36 h *p.p.* in the PDS+ sows [55]. The homeostatic function of CgA in humans includes the endocrine, cardiovascular, and immune systems, and the glucose and calcium balances [60]. Salivary CgA is regulated by a neuronal pathway [61] and is a reliable marker of stress in humans [62, 63]. In the human gastrointestinal tract, CgA is released from enterochromaffin cells and from neurons of submucosal and myenteric ganglia [64, 65], and may modulate colonic motility in response to inflammation [66]. To our knowledge, CgA has never been studied in the porcine gastrointestinal tract, but the present results warrant further investigation, since constipation is considered to be a major feature of PDS [67].

The higher serum and salivary cortisol and higher serum CgA concentrations demonstrated in the PDS+ sows may reflect differences in stress level [40] in the two groups, but could also be related to inflammation caused for example by a bacterial infection [20]. CgA has been investigated to a limited extent in swine, but a previous study demonstrated CgA release in response to experimentally induced transportation stress [40]. To determine the potential occurrence of stress in PDS+ sows, detailed behavioral observations are needed.

An increased cortisol level during the periparturient period is a normal physiological occurrence [15, 16] as also suggested by the results in our study, where PDS- sows showed increased cortisol concentrations (compared to the baseline) from − 24 h *a.p.* (Table 1). Inflammation [20] and stress [18] have also been shown to cause cortisol release in pigs. We have previously demonstrated a significant inflammatory response 12–36 h *p.p.* in the PDS+ sows [55], which could have contributed to the higher cortisol concentrations observed in sows developing PDS. Normal physiological alterations, inflammation, and stress may all have contributed to the increased cortisol concentration observed in the PDS+ sows, but stress was not further assessed in our study populations.

### 8-epi-PGF_2α_

The increased 8-epi-PGF_2α_ concentrations in PDS+ sows suggest that oxidative stress may be a feature of PDS, caused either by antioxidant deficiency or excessive production of free radicals [42]. Cells of the immune system are particularly susceptible to oxidation [68] and oxidative stress may thus affect the immune responses. 8-epi-PGF_2α_ is released after stimulation of the adrenal cortex [69], thus explaining the coinciding 8-epi-PGF_2α_, CgA and salivary cortisol responses 0–12 h after parturition. Further research is required to determine the role of 8-epi-PGF_2α_ and its potential as an early biomarker for PDS.

### Glucose and C-peptide

Compared to the baseline, a significantly increased fasting blood glucose concentration was demonstrated in both groups after parturition (Table 1). Increased blood glucose and reduced insulin responsiveness have previously been described in late gestation and early lactation in healthy sows [17, 70]. Reduced insulin responsiveness is believed to support the transportation of glucose into the udder [11, 71]. The changes observed in PDS- sows (unaltered C-peptide concentration in conjunction with increased blood glucose) are consistent with the reduced insulin responsiveness normally observed in the transition period. Interestingly, three individual sows displayed extremely high glucose values ranging from 12.7 to 24.3 × 10^− 3^ mol/L (Fig. 2D). In these sows, the high glucose concentrations were accompanied by high C-peptide values (Additional file 4). A similar syndrome, referred to as “physiological insulin resistance”, is described in humans after fasting [72]. The significantly decreased levels of C-peptide in the PDS+ sows compared to the baseline may reflect insufficient feed intake.

The sow’s ability to mobilize glucose might be crucial for the development of PDS, and post-feeding blood glucose and insulin concentrations can affect piglet growth [73]. The poor litter weight gain and lower C-peptide concentration 12–24 h *p.p.* in the PDS+ sows could reflect a reduced feed intake and low lipid and protein metabolism. Indeed, glucose homeostasis is challenged during parturition, where sows may be depleted of energy if the onset of farrowing starts more than 3 h after the last meal was consumed [74]. However, we were not able to demonstrate an association between PDS and feeding time relative to parturition.

### Prolactin

Reduced prolactin concentration has previously been found in sows suffering from metritis-mastitis-agalactia syndrome [75], and experimental lipopolysaccharide (LPS) administration has also been shown to cause lowered serum prolactin [76]. In contrast to these previous studies, prolactin decreased in PDS- sows, but remained unchanged in PDS+ sows (Table 1). How prolactin may be involved in the reduced milk production observed in PDS+ sows was not clear from our results.

## Conclusions

Salivary CgA, cortisol and serum 8-epi-PGF2α are increased in PDS+ sows before parturition, reflecting a situation of activation of the adrenergic system (CgA), adrenal system (cortisol) and oxidative stress (8-epi-PGF2α), and these analytes may potentially serve as biomarkers in the early detection of PDS. In addition, PDS+ sows showed metabolic changes consisting of decreased glucose and C-peptide concentrations that may be caused by a lower energy intake due to sickness.

The interrelationship between the metabolites and hormones assessed in the current study are not entirely clear. Our results suggest that the homeostasis of PDS+ sows may be affected before parturition and that the normal periparturient glucose metabolism is disrupted in PDS+ sows. The persistently high CgA concentration in PDS+ sows is a highly interesting finding that warrants further investigation.

## Methods

### Experimental design

The study was carried out as described in detail in our previous publication [55]. Briefly, a case-cohort study was performed in one herd that bought sows from a conventional breeder. In all, 109 sows were included, from which 38 (34.9%) were categorized as PDS positive (PDS+) and retrospectively matched with the 38 healthy sows (PDS-).

All sows (*n* = 109) were systematically observed and sampled every 24 h from at least 60 h before expected parturition until PDS occurred or until 36 h *p.p*. Due to animal welfare considerations it was necessary to treat sick sows. Therefore, a clinical definition of PDS were defined and sows was deemed PDS+ if at least two of the following clinical criteria were fulfilled: 1. Reduced feed intake, defined as “trough not empty within 30 min after feeding”, 2. General inflammation of the udder, characterized by a subjective assessment of redness, swelling and increased skin temperature, 3. rectal temperature ≥ 39.5 °C. Sows farrowing prematurely or sows that were treated for other reasons were excluded from the study. All observations were performed by a veterinarian (the first author). The sows remained in the farm and were kept according to common management routines after completion of the study.

Prior to parturition, monitoring included: 1. samples of saliva and capillary blood taken before the morning feeding as described below, 2. clinical examinations and 3. blood sampling from *v. jugularis* as described below. Except for 1., all recordings were done after the morning feeding.

Body condition was evaluated based on a Danish 4-point scoring system, where score was 1 considered thin, 2) lean, 3) medium and optimal body condition, and 4) fat [77]. Sows that were considered PDS+ (*n* = 38) were treated immediately after the veterinary clinical examination and sampling. All sows continued in the production at the farm after they exited the study.

Litters were equalized to 15 piglets and subsequently weighed on two occasions: within 24 h *p.p*. and again when the sows left the study. The weight of dead piglets was registered daily and included in the total litter weight. Cross-fostering was not allowed after litter standardization. The electronic scale (WEDO S/N 45705, Werner Dorsch GmbH, Germany) was calibrated daily.

### Sampling

Saliva was collected by a cotton swab without additives (Salivette® Cortisol, Haunisen, Denmark). The sows were allowed to chew on the swab for 3 min. The swabs were centrifuged for 5 min at 1000×g and immediately stored on ice. The saliva was tested for CgA by a time-resolved immunofluorometric assay (TR-IFMA) previously described by Escribano and coworkers [40]. Saliva cortisol was tested by a solid-phase, competitive chemiluminescent enzyme immunoassay using an automated biochemistry analyzer (IMMULITE 1000 Immunoassay System cortisol, Siemens, California, US) as previously described [18] and according to the manufacturer’s instructions. Blood sample droplets for fasting blood glucose were collected from *v. auricularis* after administration of cutaneous lidocaine spray on the dorsal area of the ear (Xylocain 100 mg/mL, AstraZeneca, UK). The blood glucose concentration was immediately measured using the Accu-Chek Aviva system (Roche Diagnostics, Basel, Switzerland; [78]). Following sampling, each sow was given a small lump of sugar as a “reward”. Blood samples were collected from *v. jugularis* in tubes without additives (BD, New Jersey, US) for preparation of serum and kept at room temperature for a maximum of 30 min. before being processed. The tubes were centrifuged for 10 min. at 3000×g and sera and saliva were stored at -80 °C until analysis. Potassium (K) and sodium (Na) were analyzed by the Hematology System Complete Blood Count method using an automated biochemistry analyzer (ADIVA 2120/2120i, Siemens Healthcare A/S, Denmark). Plasma prolactin was analyzed by a commercially available porcine ELISA kit (#SEA846Po, Cloud-Clone Corp. Texas, US) as described elsewhere [79]. The absorbance was read at 450 nm (Polar Star/Galaxy, BMG Labtech, Germany). Concentrations of C-peptide in serum were determined by a porcine-specific C-peptide ELISA (#10-1256-01, Mercodia AB, Sweden) as described previously [80–82] in accordance with the instructions given by the manufacturer, and the absorbance was read at 450 nm (Polar Star/Galaxy, BMG Labtech, Germany). The concentration of 8-epi prostaglandin F2 Alpha (8-epi-PGF2α) was analyzed by a pan-308 species commercial ELISA (#CEA701Ge, Cloud-Clone Corp., Texas, US) as described in humans by Haxhi and coworkers [83].

### Statistical analyses

The exact sampling times (date:hour:min.) were retrospectively related to the exact time of the birth of the first piglet as revealed by the video records.

The sampling time points were grouped into time intervals where 0 h was the time of birth of the first piglet: A. -60 to − 36 h; B. -36 to − 24 h; C. -24 to − 12 h; D. -12 to 0 h; E. 0 to 12 h; F. 12 to 24 h, and G. 24 to 36 h. The number of observations within each interval may vary because of the individual sampling times relative to parturition (0 h).

For statistical evaluation, two autoregressive linear regression models (A and B) were performed in the PROC MIXED procedure of Statistic Analytical Software, Enterprise Guide 7.1 (SAS® Institute, Cary, North Carolina, USA). Least-squares means (LSMEANS) and standard deviations (SD) were included in the statistical model A. Model A included OUTCOME PARAMETER_ij_ = μ + TIME_i_ + GROUP_j_ + TIME*GROUP_ij_ + ε_ij,_ where OUTCOME PARAMETER_ij_ was the measured value of the hormone or metabolic parameters; μ was the value of the observations at time 0; TIME_i_ was the explanatory variable “time intervals A-G”; GROUP_j_ was the explanatory variable “PDS+/PDS-”; TIME*GROUP_ij_ was the interaction between the two groups and time, and ε_ij_ was the random residual error term. When significant interaction was identified using model A, differences between the relevant groups and time intervals were accepted. In case of non-significant interaction, model A was replaced with model B consisting of the OUTCOME PARAMETER_ij_ = μ + TIME_i_ + GROUP_j_ + ε_ij_. If non-significant changes in TIME_i_ occurred in model B, the OUTCOME PARAMETER_ij_ was considered non-significant. Significance was considered for *p* < 0.05. Parity and body condition score were included as explanatory variables. From preliminary analyses, obstetric aid and farrowing duration were not found to be associated with any of the outcome variables. Logarithmic transformation was used for serum and saliva cortisol, CgA, prolactin, Na and K, in order to improve normality of residuals plots. The back-transformed SD for these variables is not normally distributed and cannot be interpreted directly. Fischer’s exact test was used to examine associations between PDS and body condition, obstetric aid and the possible impact of feeding time relative to parturition (less than or more than 4 h) in the PROC FREQ procedure of Statistic Analytical Software, Enterprise Guide 7.1 (SAS® Institute, Cary, North Carolina, USA). An average weight gain per hour (kg/h) was calculated for each litter. These were compared for PDS+ and PDS- piglets using the PROC MIXED procedure of Statistic Analytical Software, Enterprise Guide 7.1 (SAS® Institute, Cary, North Carolina, USA). The association between parturition duration and PDS was tested by a simple t-test in the PROC TTEST procedure of Statistic Analytical Software, Enterprise Guide 7.1 (SAS® Institute, Cary, North Carolina, USA).

## Additional files

Additional file 1: Raw data of prolactin (10^− 9^ g/L) assessed from 60 h *ante partum* (time interval A) until 36 h post partum (time interval G) in sows suffering from postpartum dysgalactia syndrome (PDS+, red) and healthy sows (PDS-, blue). Each point represents the precise sample time of each observation relative to parturition of piglet number one (0 h). The line represents the mean value at each sampling time point. (DOCX 33 kb)

Additional file 2: Raw data of sodium (Na; 10^− 3^ mol/L) assessed from 60 h *ante partum* (time interval A) until 36 h post partum (time interval G) in sows suffering from postpartum dysgalactia syndrome (PDS+, red) and healthy sows (PDS-, blue). Each point represents the precise sample time of each observation relative to parturition of piglet number one (0 h). The line represents the mean value at each sampling time point. (DOCX 35 kb)

Additional file 3: Raw data of potassium (K; 10^− 3^ mol/L) assessed from 60 h *ante partum* (time interval A) until 36 h post partum (time interval G) in sows suffering from postpartum dysgalactia syndrome (PDS+, red) and healthy sows (PDS-, blue). Each point represents the precise sample time of each observation relative to parturition of piglet number one (0 h). The line represents the mean value at each sampling time point. (DOCX 31 kb)

Additional file 4: Glucose and C-peptide concentrations obtained in three sows on repeated sampling occasions. (DOCX 14 kb)

## Abbreviations

8-epi-PGF2α: 8-epi prostaglandin F2 Alpha
*a.p.*: *Ante partum*
CgA: Chromogranin A
K: Potassium
LPS: Lipopolysaccharide
LSMEANS: Least-squares means
Na: Sodium
*p.p.*: *Post partum*
PDS: Postpartum dysgalactia syndrome (PDS+ is cases and PDS- is healthy sows)
SAM: Sympathico-adrenal medullary system
SD: Standard deviation
