# Supplementary figures and images for: Hormonal and metabolic indicators before and after farrowing in sows affected with postpartum dysgalactia syndrome
Source: BMC Vet Res. 2018 Nov 7;14:334. doi: 10.1186/s12917-018-1649-z (PMC6223068; doi:10.1186/s12917-018-1649-z)

Prolactin 10-9 g/L


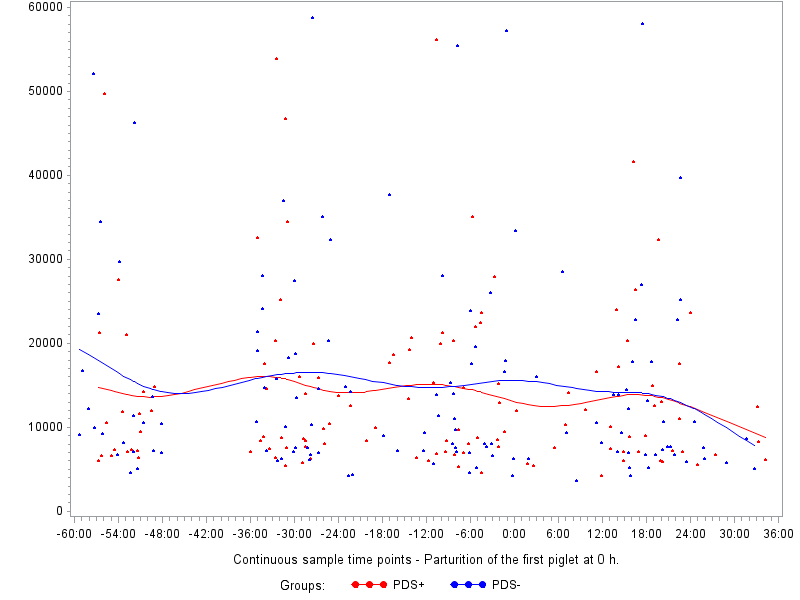


A B C D E F G

Supplement: Supplementary file 1 — Raw data of prolactin (10− 9 g/L) assessed from 60 h ante partum (time interval A) until 36 h post partum (time interval G) in sows suffering from postpartum dysgalactia syndrome (PDS+, red) and healthy sows (PDS-, blue). Each point represents the precise sample time of each observation relative to parturition of piglet number one (0 h). The line represents the mean value at each sampling time point. (DOCX 33 kb) [file 12917_2018_1649_MOESM1_ESM.docx]

Na 10-3 mol/L


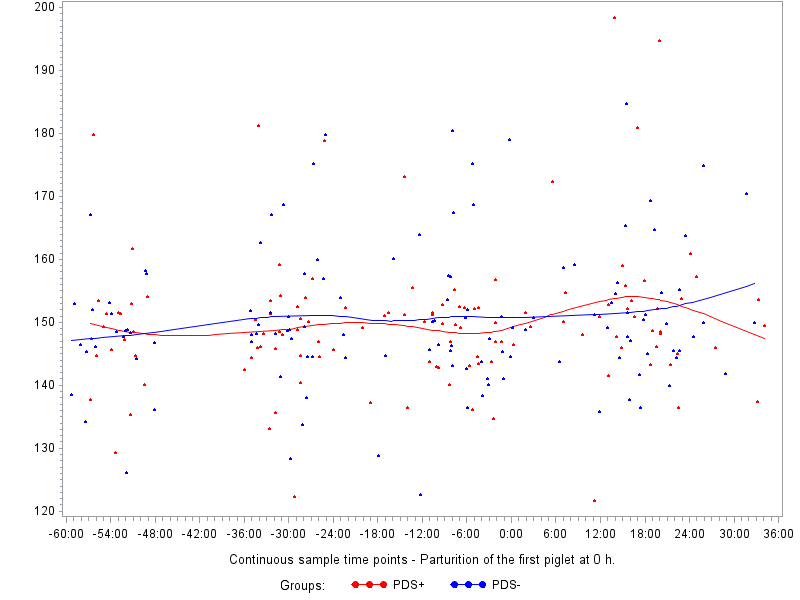


A B C D E F G

Supplement: Supplementary file 2 — Raw data of sodium (Na; 10− 3 mol/L) assessed from 60 h ante partum (time interval A) until 36 h post partum (time interval G) in sows suffering from postpartum dysgalactia syndrome (PDS+, red) and healthy sows (PDS-, blue). Each point represents the precise sample time of each observation relative to parturition of piglet number one (0 h). The line represents the mean value at each sampling time point. (DOCX 35 kb) [file 12917_2018_1649_MOESM2_ESM.docx]

K 10-3 mol/L


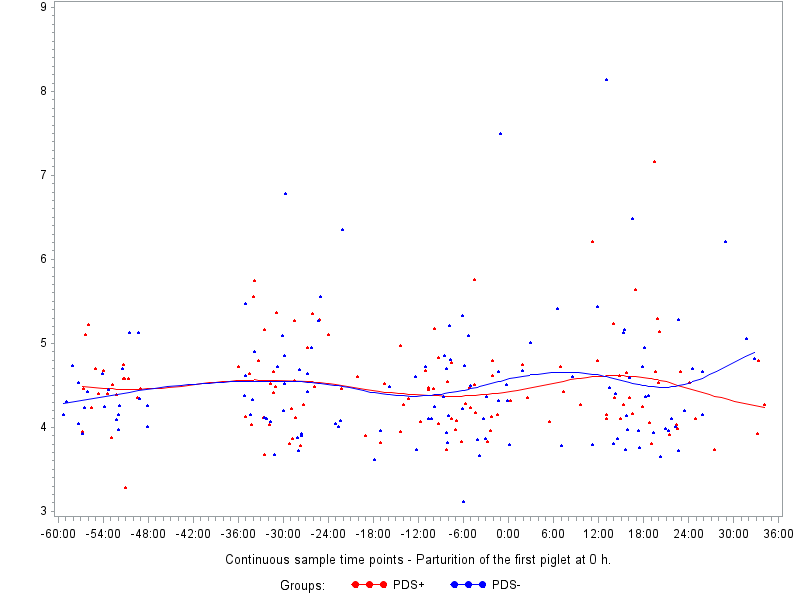


A B C D E F G

Supplement: Supplementary file 3 — Raw data of potassium (K; 10− 3 mol/L) assessed from 60 h ante partum (time interval A) until 36 h post partum (time interval G) in sows suffering from postpartum dysgalactia syndrome (PDS+, red) and healthy sows (PDS-, blue). Each point represents the precise sample time of each observation relative to parturition of piglet number one (0 h). The line represents the mean value at each sampling time point. (DOCX 31 kb) [file 12917_2018_1649_MOESM3_ESM.docx]
