# Supplementary material for: Hormonal and metabolic indicators before and after farrowing in sows affected with postpartum dysgalactia syndrome
Source: BMC Vet Res. 2018 Nov 7;14:334. doi: 10.1186/s12917-018-1649-z (PMC6223068; doi:10.1186/s12917-018-1649-z)
Supplement: Supplementary file 4 — Glucose and C-peptide concentrations obtained in three sows on repeated sampling occasions. (DOCX 14 kb) [file 12917_2018_1649_MOESM4_ESM.docx]

| Sow number | Date | Fasting blood glucose  10^-3^ mol/L | C-peptid  10^-12^ mol/L |
| --- | --- | --- | --- |
| 76 | 05 April 2014 | 12.7 | 425.62 |
|  | 06 April 2014 | 17.2 | 766.21 |
|  | 07 April 2014 | 24.3 | Missing |
| 1314 | 06 April 2014 | 19.6 | 393.49 |
| 1471 | 05 April 2014 | 14 | 523.57 |
